# DFT-based study of the structural, optoelectronic, mechanical and magnetic properties of Ti_3_AC_2_ (A = P, As, Cd) for coating applications

**DOI:** 10.1039/d1ra07856a

**Published:** 2022-02-03

**Authors:** R. M. Arif Khalil, Muhammad Iqbal Hussain, Nadia Luqman, Fayyaz Hussain, Anwar Manzoor Rana, Muhammad Saeed Akhtar, Rana Farhat Mehmood

**Affiliations:** Materials Simulation Research Laboratory (MSRL), Department of Physics, Bahauddin Zakariya University Multan 60800 Pakistan miqbal@ue.edu.pk fayyazhussain248@yahoo.com; Department of Physics, University of Education Lahore 54000 Pakistan saeed.akhtar@ue.edu.pk; Department of Chemistry, University of Education Lahore 54000 Pakistan

## Abstract

The first-principles approach has been used while employing the Perdew–Burke–Ernzerhof exchange-correlation functional of generalized gradient approximation (PBE-GGA) along with the Hubbard parameter to study the structural, optoelectronic, mechanical and magnetic properties of titanium-based MAX materials Ti_3_AC_2_ (A = P, As, Cd) for the first time. As there is no band gap found between the valence and conduction bands in the considered materials, these compounds belong to the conductor family of materials. A mechanical analysis carried out at pressures of 0 GPa to 20 GPa and the calculated elastic constants *C*_*ij*_ reveal the stability of these materials. Elastic parameters, *i.e.*, Young's, shear and bulk moduli, anisotropy factor and Poisson's ratio, have been investigated in the framework of the Voigt–Reuss–Hill approximation. The calculated values of relative stiffness are found to be greater than ½ for Ti_3_PC_2_ and Ti_3_AsC_2_, which indicates that these compounds are closer to typical ceramics, which possess low damage tolerance and fracture toughness. Optical parameters, *i.e.*, dielectric complex function, refractive index, extinction coefficient, absorption coefficient, loss function, conductivity and reflectivity, have also been investigated. These dynamically stable antiferromagnetic materials might have potential applications in advanced electronic and magnetic devices. Their high strength and significant hardness make these materials potential candidates as hard coatings.

## Abstract 2

The first-principles approach has used the Perdew–Burke–Ernzerhof exchange-correlation functional of generalized gradient approximation along with the Hubbard parameter to study various properties of titanium-based MAX materials Ti_3_AC_2_ (A = P, As, Cd).

## Graphical abstract

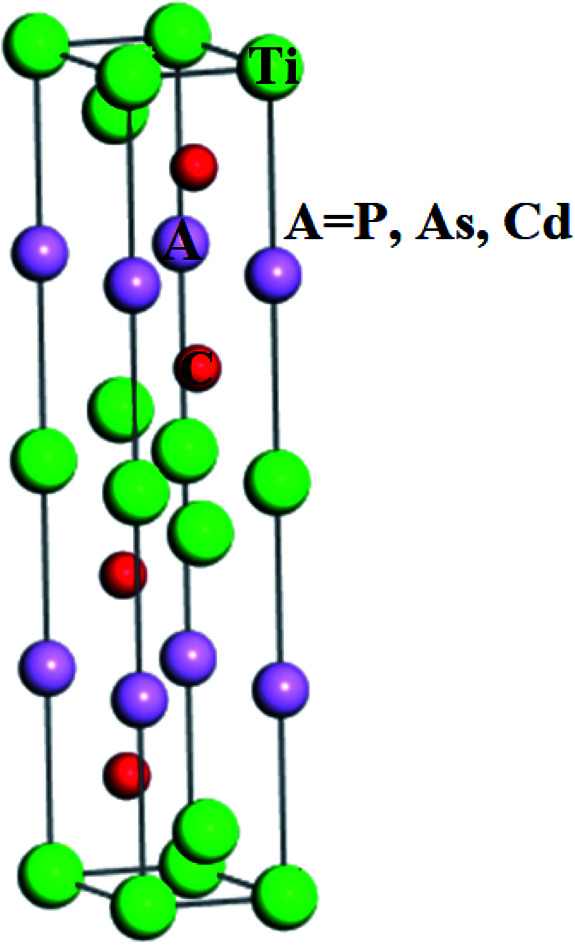

## 1. Introduction

Recently, MAX phases have captured the attention of a lot of researchers due to the discovery of MXenes,^1–5^ which are a novel 2D material made from transition metal carbides and nitrides. MXenes offer higher shielding effectiveness due to their moderate electrical conductivity and can be obtained by the selective etching of the A-layer from a MAX material.^6,7^ MXenes have the capability to provide perfect guidance to design shielding materials for electromagnetic interference,^8^ and can be used as an electrode material to enhance the capacitance of super-capacitors.^9^ The family of MAX materials exhibiting properties of metals and ceramics simultaneously was first reported in the early 1960s by Nowtony and co-workers. Later, in 1996, Barsoum *et al.* discovered a fascinating MAX material, titanium silicon carbide T_3_SiC_2_.^10–13^ Titanium-based MAX materials like Ti_3_SiC_2_, Ti_3_AlC_2_ and Ti_2_AlC have demonstrated unique properties such as high temperature strength, low density and good oxidation resistance.^14,15^ Moreover, lithium-based MAX materials like Li_2_Ti_3_O_7_, LiNiO_2_, Li_*x*_CoO_2_, and Li_*x*_TiO_2_ have extraordinary technological applications.^16,17^

MAX materials are denoted as M_*n*+1_AX_*n*_, where M represents a transition metal, A represents an element from groups XIII–XVI, X is either carbon or nitrogen and *n* may vary from 1–3.^18,19^ MAX compounds are categorized in distinct phases, namely, 211, 312 and 413, with respect to the value of *n*.^20^ The foremost distinction among MAX alloys depends upon the number of inserting A layers per M layers.^21^ Mechanically, MAX compounds are different from MX carbides and nitrides.^22^ The incredible characteristics of MAX phases solely depend on M–X bonds with covalent–metallic nature, and are extremely strong when compared with M–A bonds.^23^ Ti_3_AlC_2_ from the Ti–Al–C family is a very interesting MAX material due to its greatly tailorable properties.^24^ Experimental and computational investigations to explore the properties of MAX phase materials have been reported. Various MAX materials like Ti_3_SiC_2_, Zr_2_AlC_2_, V_2_AlC, V_4_AlC_3−*x*_, V_12_Al_3_C_8_, Mo_2_TiAlC_2_, Mo_2_Ti_2_AlC_3_, Ti_3_AlC_2_, Ti_2_InC, Zr_2_InC and Hf_2_InC have demonstrated unique and promising structural, mechanical, electrical and optical properties.^25–31^ MAX phases have great importance in shielding and coating applications, such as the *in situ* growth of MAX phase coatings on carbonised wood and their terahertz shielding properties,^32^ exfoliation and defect control of the two-dimensional few-layer MXene Ti_3_C_2_T_*x*_ for electromagnetic interference shielding coatings,^33^ highly conductive and scalable Ti_3_C_2_T_*x*_-coated fabrics for efficient electromagnetic interference shielding,^34^ “beyond Ti_3_C_2_T_*x*_: MXene for electromagnetic interference shielding”^35^ and MAX phase-based electroconductive coating for high-temperature oxidizing environment.^36^

As per the literature, there is no experimental or computational study reported till date on the novel Ti_3_AC_2_ (A = P, As, Cd) combination of titanium-based MAX materials. Since MAX phases have great importance in shielding and coating applications, as discussed above, this challenge has motivated us to computationally inspect the structural, optoelectronic, mechanical and magnetic properties of these compounds for the first time using an *ab initio* approach, where calculations have been performed using the CASTEP simulation code.

## 2. Computational methodology

The first-principles simulation was performed opting for the plane wave pseudo-potential based on DFT,^37^ as employed in the CASTEP code.^38^ The structural parameters were investigated by considering electron–ion interactions with a cut-off energy of 600 eV using norm conserving pseudo-potentials.^39,40^ A Monkhorst–Pack grid of 10 × 10 × 2 was chosen to model the Brillouin zone (BZ).^41^ For settling down ionic positions, the conjugate gradient method was used.^42^ The structural parameters of the system in anticipation of Hellmann–Feynman forces were found to be significantly less than 0.02 eV Å^−1^ in an energy convergence criterion of 1 × 10^−5^ eV.^43^ The PBE-GGA functional in addition to the Hubbard parameter *U* was utilized, particularly to evaluate the electronic and magnetic properties of the system.^44,45^ The values of *U* for Ti and Cd atoms were opted as 2.5 eV and 2.0 eV, respectively. The *U*-values opted for these calculations are taken from the standard parameterization, as mentioned in the CASTEP code. Optical and mechanical properties were calculated under the umbrella of Kramer–Kronig relations^46,47^ and the Voigt–Reuss–Hill approximation.^48,49^ To determine the thermal stability of Ti_3_AC_2_ (A = P, As, Cd), phonon frequencies have been illustrated while utilizing density functional perturbation theory.^50^

## 3. Results and discussions

### 3.1. Structural and electronic properties

**Fig. 1:**
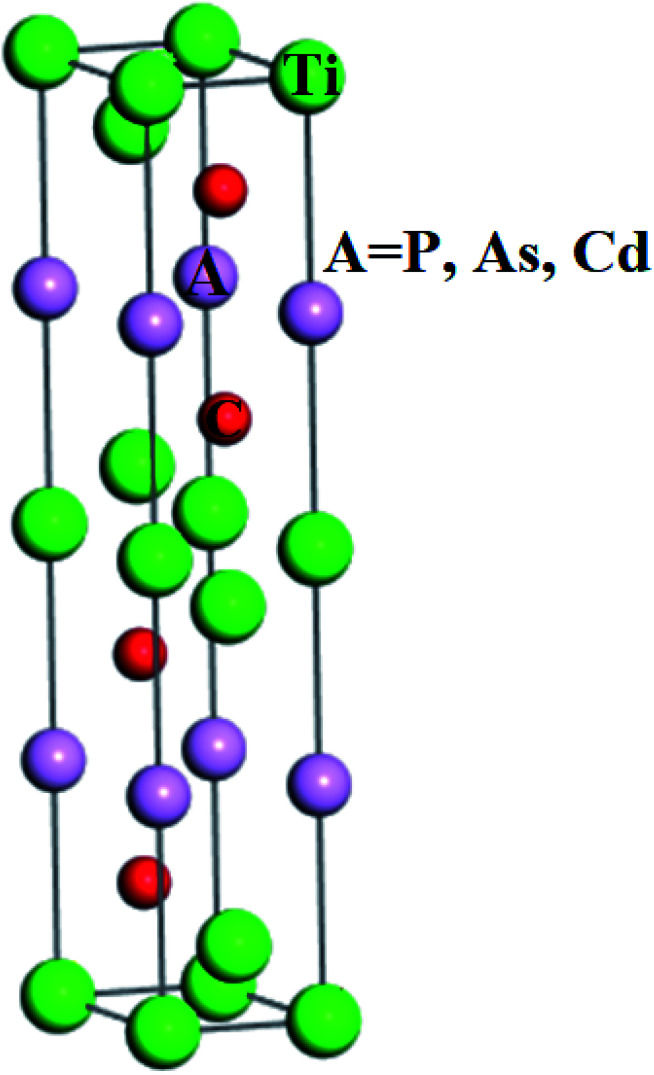
The crystal structure of Ti_3_AC_2_ (A = P, As, Cd).

The structural investigations revealed that Ti_3_AC_2_ (A = P, As, Cd) exhibited a hexagonal crystal structure with the point group (*D*_6h_, 6/*mmm*, 6/*m* 2/*m* 2/*m*) and space group (*P*6_3_/*mmc*, *P*6̄*c*2*c*). Interestingly, the structure of Ti_3_AC_2_ (A = P, As, Cd) is found to be similar to many MAX materials, such as Ti_3_SiC_2_ and Ti_3_GeC_2_,^24,51^ with its lattice parameters given in Table 1. The Wyckoff positions of Ti, A and C atoms are located at 2a, 2b and 4f, respectively, as shown in Fig. 1.

**Table 1:** Calculated structural parameters for the Ti_3_AC_2_ (A = P, As, Cd) compounds

Material	*a* (Å)	*c* (Å)	*c*/*a*	*V* (Å^3^)	*E*_0_ (Ry)
Ti_3_PC_2_	3.149	16.798	5.33	144.284	−11 919.74
Ti_3_AsC_2_	3.149	16.798	5.33	144.284	−19 595.75
Ti_3_CdC_2_	3.167	18.906	5.969	164.276	−32 935.30

**Fig. 2:**
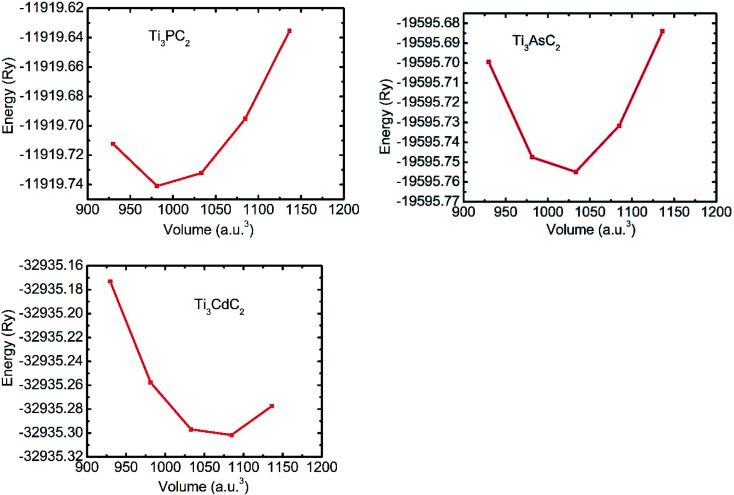
Energy *vs.* volume optimization graphs of Ti_3_PC_2_, Ti_3_AsC_2_ and Ti_3_CdC_2_.

The search for geometric optimization and structural stability is the initial step in any first-principles simulation. For this reason, energy *versus* volume graphs for each Ti_3_AC_2_ (A = P, As, Cd) material are plotted in Fig. 2, and the data is fitted rendering to the rule of energy of state (EOS) owing to Birch–Murnaghan.^52^ The calculated formation energy of the considered Ti_3_AC_2_ (A = P, As, Cd) materials is negative, which indicates the structural stability of these MAX materials.^53^ The negative ground state energy values for Ti_3_AC_2_ (A = P, As, Cd) at static equilibrium are found to be −11 919.74 Ry, −19 595.75 Ry and −32 935.30 Ry, respectively.

**Fig. 3:**
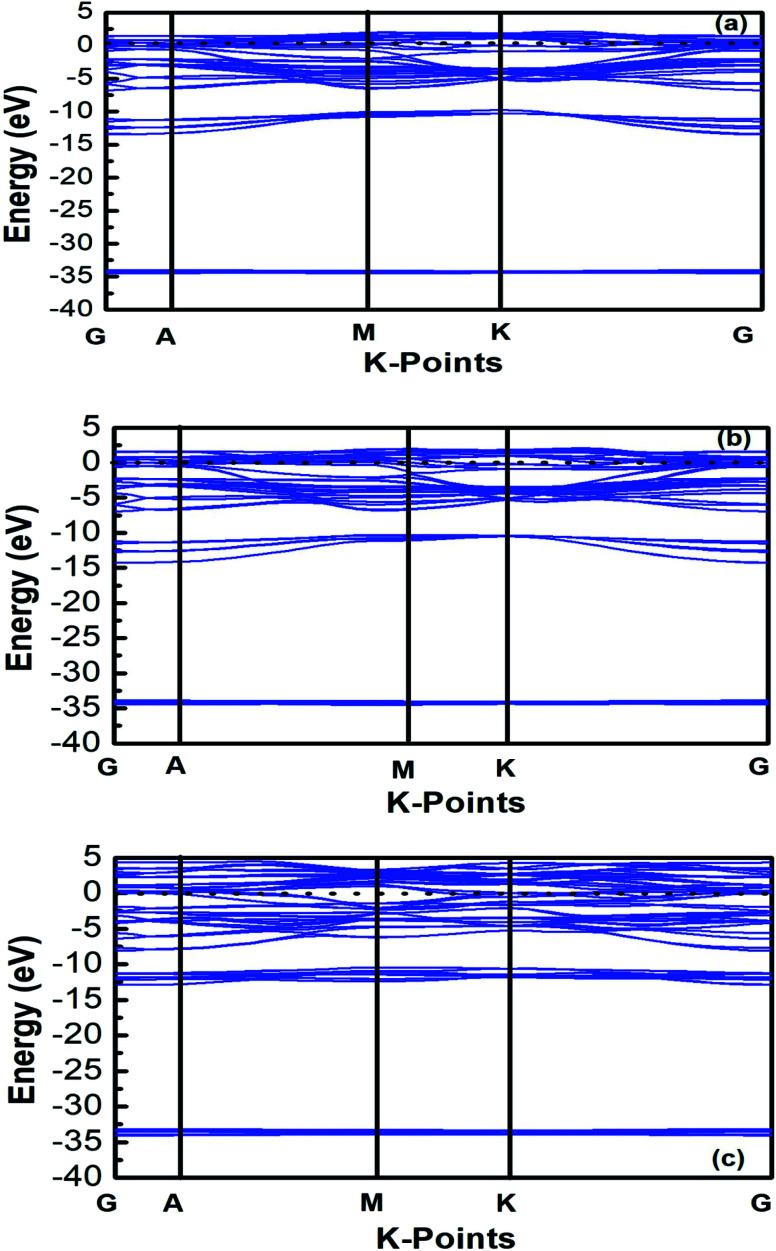
Band structures of (a) Ti_3_PC_2_, (b) Ti_3_AsC_2_ and (c) Ti_3_CdC_2_.

**Fig. 4:**
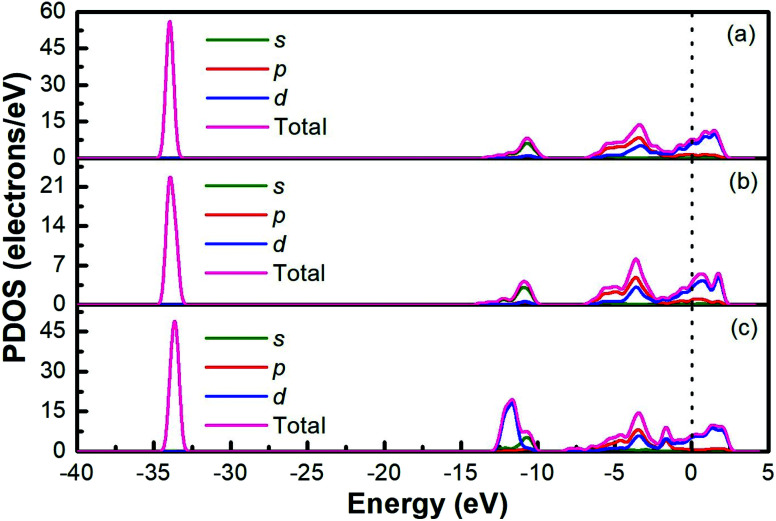
Partial density of states of (a) Ti_3_PC_2_, (b) Ti_3_AsC_2_ and (c) Ti_3_CdC_2_.

The band structures of Ti_3_AC_2_ (A = P, As, Cd) have been anticipated within the 1st Brillouin zone (BZ) along with high symmetry lines from the calculated structural parameters. Fig. 3 depicts the electronic band structures of Ti_3_PC_2_, Ti_3_AsC_2_ and Ti_3_CdC_2_, and reveals the metallic behaviour of these compounds. As a matter of fact, no band gap has appeared across the Fermi level (*E*_F_), and the electronic states of valence and conduction bands are overlapping. Such metallic behaviour of the Ti_3_AC_2_ compounds is quite analogous to a few already reported materials with MAX phases.^54,55^ Ti_3_AC_2_ (A = P, As, Cd) can offer excellent electrical, thermal and metallic conductivity. The total density of states (TDOS) of Ti_3_PC_2_, Ti_3_AsC_2_ and Ti_3_CdC_2_ determined at *E*_F_ revealed that these materials exhibit 5.87, 6.22 and 2.95 states per eV, respectively, as depicted in Fig. 4 along with the PDOS. Moreover, it has been observed that the value of TDOS for Ti_3_AsC_2_ is slightly greater than those of Ti_3_PC_2_ and Ti_3_CdC_2_, indicating the more conductive nature of this compound. The occupied valence states of Ti_3_PC_2_, Ti_3_AsC_2_ and Ti_3_CdC_2_ are observed to be at −6.13, −6.00 and −8.35 eV, respectively, with respect to the *E*_F_, as shown in Fig. 3.

Fig. 4(a) demonstrates the partial density of states (PDOS) for Ti_3_PC_2_. Hybridization in the valence band from −11.6 to −9.7 eV occurs due to the 3s states of P and 2s states of C. The hybridization between the 3d states of Ti, 3p states of P and C in the valence band from −4.2 to −2.0 eV resulted in raising the top of the valence band towards the *E*_F_. As for the conduction band, the contribution of the 3d states of Ti near the *E*_F_ appears as a higher density of states in the region from 1.4 to 2.8 eV. Fig. 4(b) demonstrates the PDOS for Ti_3_AsC_2_. Hybridization of the s states of As and C appeared from −10.6 to 9.6 eV. The top of the valence band rises towards the *E*_F_ due to the mix p states of As, C and the d states of Ti in the valence band from −3.6 to −1.6 eV. The maximum density of states in the conduction band appears due to the 3d states of Ti from 1.9 to 2.7 eV. Fig. 4(c) shows that the hybridization in Ti_3_CdC_2_ resulted from the 2s states of C with the 3p and 3d states of Ti from −11.5 to −9.5 eV. The maximum density of states within the valence band appears due to the 3d states of Cd from −12.6 to −10.7 eV. The interaction between the s and p states of Ti and C from −7.9 to −5.8 eV raises the valence band towards the *E*_F_. The strong hybridization between the 3d states of Ti and 2p states of C from −4.2 to −2.4 eV is evidence for Ti–C covalent bonds in Ti_3_CdC_2_. The maximum density of states in the conduction band from 1.5 to 2.4 eV appeared due to the 3d states of Ti.^56^

### 3.2. Optical and dynamical properties

#### 3.2.1 Optical properties

It is well known that optical properties of materials have strong correlations with their electronic band structure.^56^ The optical properties of the Ti_3_AC_2_ (A = P, As, Cd) compounds were calculated with a Gaussian smearing of 0.5 eV and variation in energy from 0 to 20 eV. The interaction of electromagnetic (EM) radiation is supposed to be plane polarized along [1 0 0] with the material and the optical parameters, *i.e.*, refractive index, extinction coefficient, absorption, loss function, optical conductivity and reflectivity, have thus been investigated.^57^

##### 3.2.1.1 Complex dielectric function

**Fig. 5:**
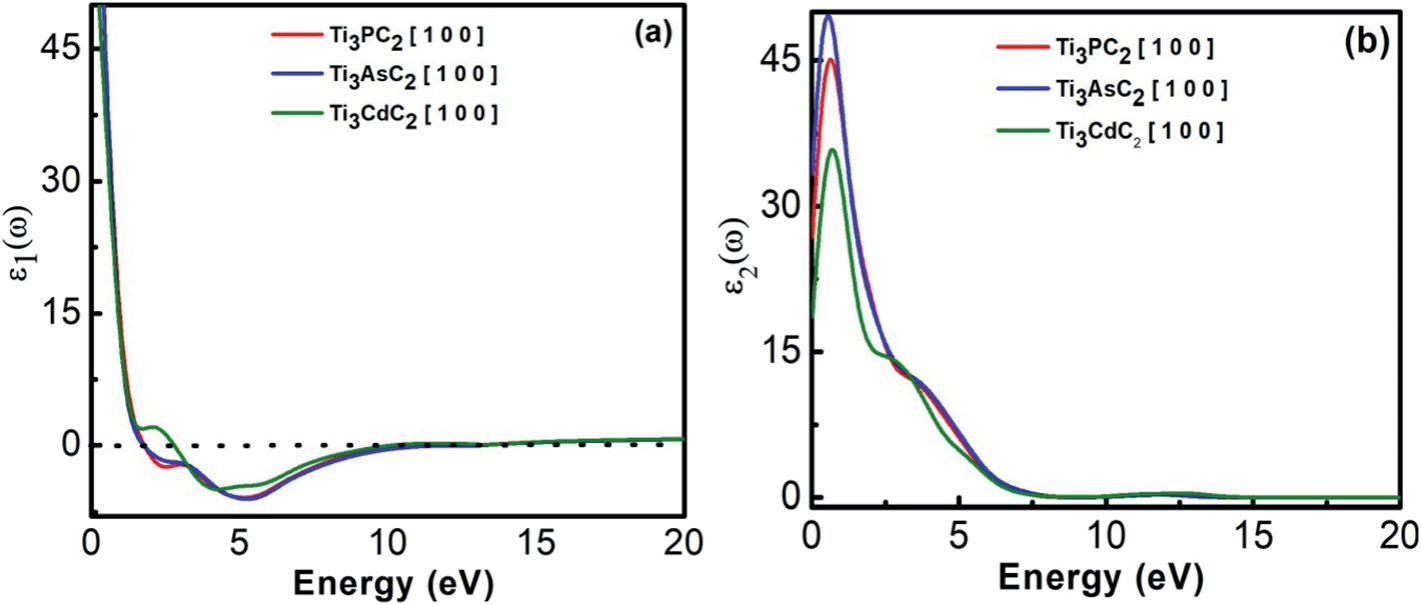
(a) Real dielectric function and (b) imaginary dielectric function of Ti_3_AC_2_ (A = P, As, Cd).

The complex dielectric constant is an energy dependent function that has major contributions towards the optical properties of materials. It has real (*ε*_1_(*ω*)) and imaginary parts (*ε*_2_(*ω*)) that provide information regarding the polarization/dispersion and absorption of light, respectively, in relation to the electronic band structure of materials. In Fig. 5(a), behaviour of the real part of the dielectric constant is depicted as a function of energy with static values of 182, 242 and 72 for Ti_3_PC_2_, Ti_3_AsC_2_ and Ti_3_CdC_2_, respectively. For all compounds, the real part of the dielectric function extends towards negative values for higher energies, showing the metallic behaviour of the compounds. Fig. 5(b) shows the behaviour of the imaginary part of the dielectric function for Ti_3_PC_2_, Ti_3_AsC_2_ and Ti_3_CdC_2_ with static values of 57, 76 and 12 at 0 eV, respectively. The imaginary part also demonstrated the metallic behaviour of the MAX compounds.

##### 3.2.1.2 Refractive index and extinction coefficient

**Fig. 6:**
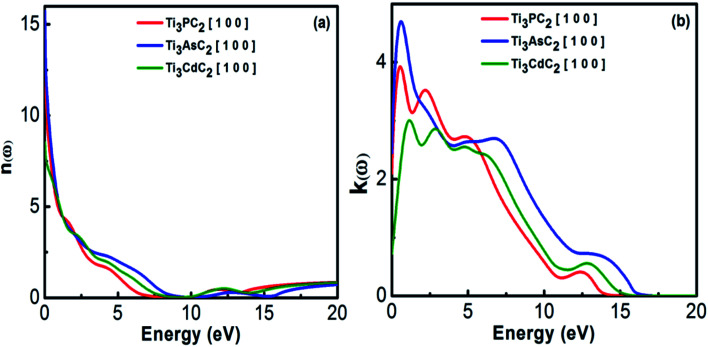
(a) Refractive index and (b) extinction coefficient of Ti_3_AC_2_ (A = P, As, Cd).

The refractive index represents the phase velocity and the extinction coefficient describes the absorption losses when EM radiation passes through the material. Fig. 6(a) shows the variation in refractive index for Ti_3_PC_2_, Ti_3_AsC_2_ and Ti_3_CdC_2_ as a function of photon energies. It has been observed that Ti_3_AsC_2_ demonstrates the maximum value of refractive index among the studied compounds due to considerable interaction between the valence electrons and incident photons. This interaction ultimately resulted in polarization within the material.^58^

Fig. 6(b) represents the static values of the extinction coefficient calculated for Ti_3_CdC_2_, Ti_3_PC_2_ and Ti_3_AsC_2_, which are found to be 0.7, 2 and 2.4, respectively. Ti_3_PC_2_ shows two strong peaks at 0.5 and 2.2 eV, Ti_3_AsC_2_ only shows a single peak at 0.7 eV, and Ti_3_CdC_2_ shows two peaks at 1.2 and 2.9 eV. The extinction coefficients for the three considered materials follow a decreasing trend for energies ranging from 5 to 20 eV. Among these three materials, the values of the extinction coefficients allude to the fact that Ti_3_AsC_2_ absorbs more radiation as compared to Ti_3_PC_2_ and Ti_3_CdC_2_.

##### 3.2.1.3 Absorption coefficient and energy loss function

**Fig. 7:**
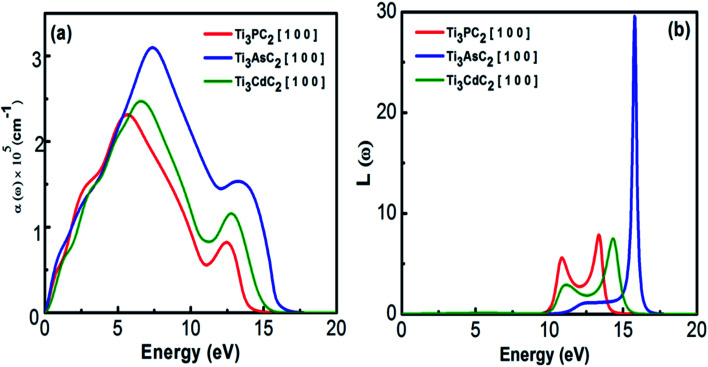
(a) Absorption coefficient and (b) energy loss function of Ti_3_AC_2_ (A = P, As, Cd).

It is well known that the absorption coefficient of any material dictates the amount of photon energy absorbed. Instead of absorption, the extinction coefficient helps to estimate the conversion efficiency of many optical materials for applications in solar devices.^59^ The absorption spectra of Ti_3_PC_2_, Ti_3_AsC_2_ and Ti_3_CdC_2_, as shown in Fig. 7(a), reveal the metallic behaviour of the compounds due to the absorption of incident photons of all energies. A sharp increasing trend in the absorption coefficient has been observed from almost 5 eV, and at energies about 14 eV, it decreases drastically. Ti_3_AsC_2_ exhibits maximum absorption as a result of transitions from the p state of As and the 3d state of Ti^60^ when compared with Ti_3_PC_2_ and Ti_3_CdC_2_. The sudden drop of absorption above 13 eV might lead to plasma resonance.

The loss function describes the plasma resonance frequencies that appear due to effects like dispersion, heating and scattering. At plasma frequencies, the loss function demonstrates its maximum value. Fig. 7(b) shows the loss functions and bulk plasma frequencies for Ti_3_PC_2_, Ti_3_AsC_2_ and Ti_3_CdC_2_ to be 7.8 (13.3 eV), 29 (15.7 eV) and 7.3 (14.3 eV), respectively. At higher frequencies, the loss function for Ti_3_CdC_2_ attains its minimum value; hence, this compound could be a suitable dielectric material. If the plasma frequencies are slightly lower than that of the incident photons, the compounds are considered transparent.^59^

##### 3.2.1.4 Optical conductivity and reflectivity

**Fig. 8:**
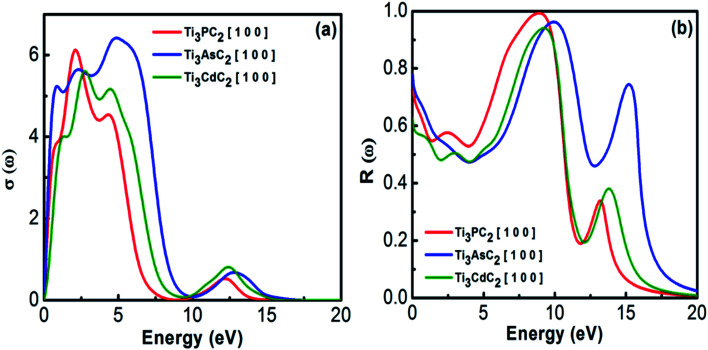
(a) Optical conductivity and (b) reflectivity of Ti_3_AC_2_ (A = P, As, Cd).

Optical conductivity describes the conductivity of EM radiation with a threshold frequency through the material's surface by inter- and intra-band transitions. It approximately follows a similar trend as that for the absorption spectra of MAX materials. Fig. 8(a) depicts the optical conductivity of the Ti_3_AC_2_ (A = P, As, Cd). The optical conductivity fluctuates between 0.2 to 7.0 eV, and attains its maximum value, *i.e.*, 2.1, 2.4 and 2.7 eV for Ti_3_PC_2_, Ti_3_AsC_2_ and Ti_3_CdC_2_, respectively, followed by a sharp decrease and a dip between 9 and 10 eV.

Reflectivity helps to explain the surface behavior of MAX materials. Reflectivity is a ratio of the energy possessed by incident photons to that of reflected photons. Maximum reflectivity is observed specifically in the UV and in the moderate IR regions. In the visible region, the considered MAX materials offer 44% reflectivity and are potential candidates to reduce solar heating.^54^ The static values of reflectivity for Ti_3_PC_2_, Ti_3_AsC_2_ and Ti_3_CdC_2_ are found to be 0.75, 0.78 and 0.63, respectively, at an incident energy of 0 eV, as shown in Fig. 8(b). A sharp increasing trend in reflectivity towards its corresponding maximum values at 8.9 eV (0.99), 10 eV (0.99), and 9.2 eV (0.94) has been observed. Afterwards, sharp and spiky dips appeared around 12 eV followed by further enhancement in the reflectivity values.

#### 3.2.2 Dynamical properties

It is worth mentioning that neither theoretical nor experimental efforts have been made so far to explore the dynamical properties of these materials. Therefore, at present, a comparative study of the observed results with the literature is not possible. However, the outcomes pertaining to calculated phonon frequencies could be beneficial for determining the dynamical and thermodynamical properties of these MAX phases experimentally.

As there are 12 atoms in Ti_3_AC_2_ (A = P, As, Cd), resulting in 36 phonon branches or modes of vibration, three modes at zero frequency are recognized as acoustic modes and the remaining 33 modes are called optical modes of vibration. From these 33 optical modes of vibration, 12 modes are found to be Raman active, 9 modes are IR active and 12 calculated modes are found to be inactive modes. Our calculated total modes for the Ti_3_AC_2_ (A = P, As, Cd) phases are consistent with a previous theoretical study of the lattice dynamics of Al-containing carbides M_3_AlC_2_ (M = Ti, V, Ta).^61^ However, a select 7 (out of 12) Raman and 6 (out of 9) IR active modes for each phase are given in Table 2, and the remaining Raman and IR active modes can be called degenerate modes, as mentioned above. These compounds are found to be dynamically stable because no imaginary frequency or soft mode^51,57^ appears at the gamma (*Γ*) point. The calculated symmetries and phonon frequencies for the considered compounds, *i.e.*, Ti_3_AC_2_ (A = P, As, Cd), are listed in Table 2. For these compounds, the modes of vibration are categorized by irreducible representations^62^ of the point group symmetry *D*_6h_ 6/*mmm* and the space group symmetry *P*6_3_/*mmc* in the hexagonal phase of the crystal structure. The highest frequencies of the Raman modes are observed at 621, 656 and 644 cm^−1^, while the highest IR modes are observed at 542, 569 and 603 cm^−1^ for Ti_3_PC_2_, Ti_3_AsC_2_ and Ti_3_CdC_2_, respectively. The modes in the range of frequency between 175 cm^−1^ to 546 cm^−1^ are due to the similar motion of Ti and C atoms in all the structures. The highest frequency modes are due to the motion of carbon atoms. The low frequency Raman modes around 98 cm^−1^ are due to the motion of the heavy atoms Ti and As in the Ti_3_PC_2_ and Ti_3_AsC_2_ phases, respectively. However, the low frequency Raman modes at 55 cm^−1^ are due to the motion of the Cd atom in the Ti_3_CdC_2_ system.

**Table 2:** Phonon frequencies (cm^−1^) of Ti_3_AC_2_ (A = P, As, Cd) calculated at the *Γ* point

Compounds	Raman	Irreducible representation	IR	Irreducible representation
Ti_3_PC_2_	97.954	E_2g_	224.587	E_1u_
	175.920	E_1g_	281.274	E_1u_
	257.668	A_1g_	327.291	A_2u_
	282.717	E_2g_	427.818	A_2u_
	549.843	E_2g_	505.430	A_2u_
	557.923	E_1g_	542.772	E_1u_
	621.215	A_1g_	—	—
Ti_3_AsC_2_	97.666	E_2g_	165.367	E_1u_
	195.099	E_1g_	254.128	A_2u_
	210.859	E_2g_	264.557	E_1u_
	292.288	A_1g_	387.196	A_2u_
	586.373	E_2g_	545.675	A_2u_
	590.789	E_1g_	569.994	E_1u_
	656.223	A_1g_	—	—
Ti_3_CdC_2_	54.925	E_2g_	82.951	E_1u_
	172.088	E_1g_	133.859	A_2u_
	175.877	E_2g_	279.249	E_1u_
	244.316	A_1g_	357.470	A_2u_
	598.555	E_1g_	540.045	A_2u_
	599.4584	E_2g_	603.724	E_1u_
	644.810	A_1g_	—	—

### 3.3. Mechanical properties

After experiencing external stress, a material's response may correspond to its elastic properties, *i.e.*, bond strength and mechanical stability. The mechanical properties of Ti_3_AC_2_ (A = P, As, Cd) at various pressure values, *i.e.*, 0 GPa, 5 GPa and 10 GPa, are listed in Tables 3 and 4. Shear deformation can illustrate the ductility of a material.^63^ Hooke's law, fitted to shapeless crystals, has been used in the framework of the robust CASTEP simulation code along with the stress–strain behavior in order to calculate the elastic parameters. As reported, the strain pattern of the materials can be reduced significantly for crystals with a low symmetry having mutually independent elastic constants.^64^

**Table 3:** Elastic constants *C*_*ij*_ of Ti_3_AC_2_ (A = P, As, Cd)

Compounds	Pressure (GPa)	*C*_11_	*C*_12_	*C*_13_	*C*_33_	*C*_44_
Ti_3_PC_2_	0	318	237	58	382	197
	5	335	105	63	365	118
	10	297	95	111	348	184
Ti_3_AsC_2_	0	340	62	79	368	146
	5	343	67	84	367	148
	10	347	71	80	376	147
Ti_3_CdC_2_	0	354	135	211	265	140
	5	355	135	202	263	137
	10	392	125	240	256	176

**Table 4:** Young's, bulk and shear moduli, Pugh's ratio, machinability index, Poisson's ratio, shear anisotropic factor, and linear compressibility coefficients of Ti_3_AC_2_ (A = P, As, Cd)

Compounds	*E* (GPa)	*B* (GPa)	*G* (GPa)	*G*/*B*	*B*/*G*	*B*/*C*_44_ (GPa)	*υ*	*A*	*α*
Ti_3_PC_2_	275	157	114	0.7	1.37	0.79	0.2	1.3	1.3
	282	155	118	0.8	1.31	0.82	0.2	0.8	1.0
	286	151	121	0.8	1.27	0.82	0.2	1.7	0.1
Ti_3_AsC_2_	326	162	140	0.9	1.16	1.10	0.2	1.1	0.8
	327	166	140	0.8	1.19	1.12	0.2	1.1	0.8
	327	166	141	0.9	1.18	1.12	0.2	1.1	0.9
Ti_3_CdC_2_	206	217	77	0.4	2.82	1.55	0.3	2.8	1.2
	184	211	68	0.3	3.10	1.54	0.3	2.5	1.4
	186	229	68	0.2	4.40	1.30	0.4	4.1	2.5

The elastic constants of the considered MAX materials satisfy Born's criterion^57^ with positive values, leading to the assessment that these materials are mechanically stable. The anisotropy factor for the hexagonal phase of MAX materials is defined as *K*_c_/*K*_a_ = (*C*_11_ + *C*_12_ − 2*C*_13_)/(*C*_33_ − *C*_13_), where *K*_c_ and *K*_a_ are the compressibility coefficients along the *c*- and *a*-axes, respectively. Bulk and shear moduli are used to determine the hardness,^65^ whereas Young's modulus is used to estimate the stiffness of solid materials.^56^ The elastic constants of the considered MAX materials reveal their anisotropic nature. Table 4 summarizes the mechanical properties of the MAX materials using the Voigt–Reuss–Hill approximation.^66–68^

The Young's (*E*), bulk (*B*) and shear (*G*) moduli can be expressed as follows:1
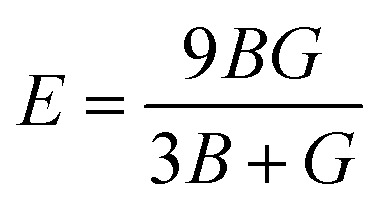
where2
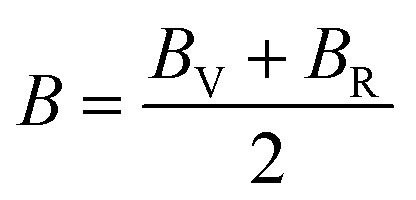
and3
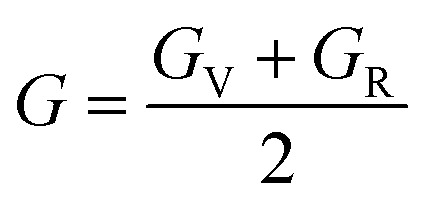


The values of Young's modulus follow the trend of Ti_3_AsC_2_ > Ti_3_PC_2_ > Ti_3_CdC_2_, revealing that Ti_3_AsC_2_ is a bit stiffer than the Ti_3_PC_2_ and Ti_3_CdC_2_ compounds. The values of the bulk modulus follow the trend of Ti_3_CdC_2_ > Ti_3_AsC_2_ > Ti_3_PC_2_. The calculated value for shear modulus follows the order of Ti_3_AsC_2_ > Ti_3_PC_2_ > Ti_3_CdC_2_. In solids, Pugh's ratios *B*/*G* and *G*/*B* decide the brittle or ductile nature of materials. If *B*/*G* > 1.75 and *B*/*G* < 0.5, then the material is considered ductile; otherwise, it is brittle.^52,58^ According to Pugh's criteria, Ti_3_PC_2_ and Ti_3_AsC_2_ possess a brittle nature, while Ti_3_CdC_2_ is found to be a ductile material. The value of the machinability index^69,70^ follows the order of Ti_3_CdC_2_ > Ti_3_AsC_2_ > Ti_3_PC_2_.

Poisson's ratio *υ*, the shear anisotropy factor *A* and the linear compressibility coefficient can be calculated using the expressions given below:4
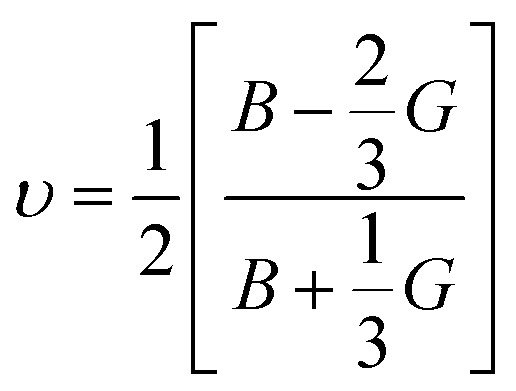
5
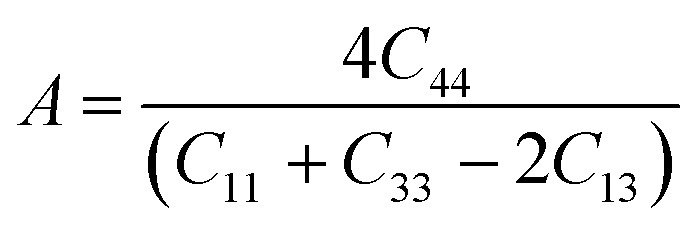
6
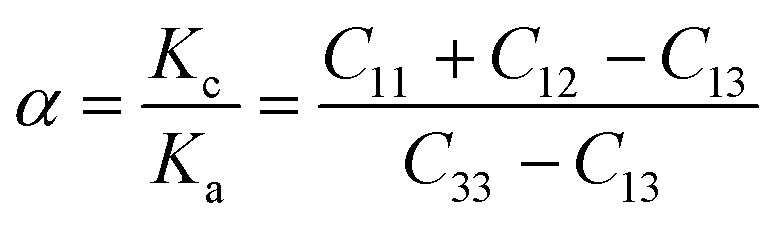


Poisson's ratio determines the degree of covalent bonding in materials.^51^ It has a value of 0.25 for ionic materials and 0.1 for covalent materials. Table 4 shows that Poisson's ratios for the studied MAX materials are around 0.25; therefore, the materials exhibit ionic character. The anisotropy factor helps to understand the isotropic or anisotropic nature of materials. If its value is 1, then the material is considered isotropic; otherwise, it is an anisotropic material.^51^ In the current study, the MAX materials are observed to be anisotropic, and might have potential applications in crystal physics and engineering sciences.^71^Table 4 reveals that the considered MAX materials exhibit large linear compressibility (*α*) along the *a*-axis instead of along the *c*-axis.^51^

#### 3.3.1 Bond stiffness

Generally, Pugh's ratio is not considered a good indicator for MAX phase materials when it comes to exhibiting their high damage tolerance and fracture toughness experimentally. Thus, the computational mode of bond stiffness can well predict the damage tolerance of these MAX phases using the ratio of bond stiffness of the weakest and strongest bonds. This criterion is also applied nowadays to ternary-layered borides such as MoAlB.^72,73^ In MAX phases, chemical bonding involves the incorporation of ionic, covalent and metallic bonding, wherein bonding between M–X slabs is deemed stronger than bonding among the M–A slabs.^22,72^ Thus, bond stiffness of the M–A slabs decreases with the increase of that of the M–X slabs in MAX phases.^73^

Furthermore, according to mechanics, for a classical spring, a relation between load and deformation is defined by Hooke's law.^73^ It is assumed here that a similar relation exists for chemical bonds in a solid, and so bond stiffness can be utilized to characterize and quantify bond strength. Specifically, the bond length *d* as a function of pressure *P* can be estimated using lattice parameters and internal coordinates. As variation in *P* causes changes in the bond strength, the relative bond lengths (*d*/*d*_0_), where *d*_0_ denotes the bond length at 0 GPa, should be linked to *P* by a quadratic curve^74^ obeying eqn (7), shown below. The slope of such a curve is defined as 1/*k*, where *k* represents the bond stiffness.^73^

7 *d*/*d*_0_ = *C*_0_ + *C*_1_*P* + *C*_2_*P*^2^

8
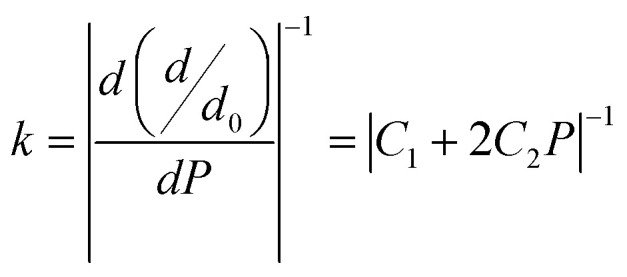
where *C*_*i*_ (*i* = 0, 1, 2) is the quadratic fitting coefficient.

**Fig. 9:**
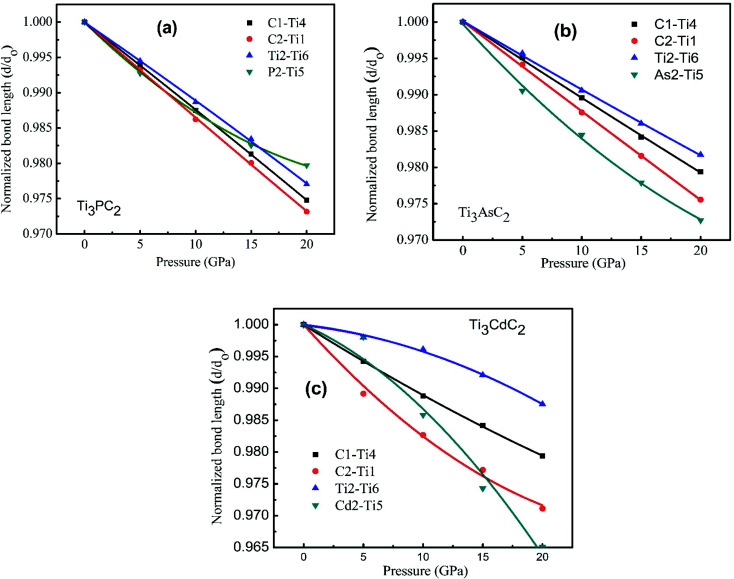
Normalized bond length as a function of the external pressure from 0 to 20 GPa for (a) Ti_3_PC_2_, (b) Ti_3_AsC_2_ and (c) Ti_3_CdC_2_.

Fig. 9(a)–(c) displays the behavior of normalized bond lengths calculated for Ti_3_AC_2_ (A = P, As, Cd) as a function of pressure from 0 GPa to 20 GPa. The declining patterns of all bonds on increasing pressure fortify our general viewpoint that bond strength increases if pressure grows systematically. Table 5 presents the various bond lengths of the considered materials, *i.e.*, Ti_3_AC_2_ (A = P, As, Cd), calculated at a diverse range of pressures, ranging from 0 GPa to 20 GPa. As expected, the variation of bond strength in the Ti_3_AC_2_ (A = P, As, Cd) compounds follows the universal change of electronic configuration due to the increase of the atomic radii of the A elements, *i.e.*, P → Cd. The results reveal that the bond length decreases with increasing pressure. In addition to this, C–Ti bonds are found to be stiffer than P–Ti bonds, while P–Ti bonds are stronger than As–Ti and Cd–Ti bonds in the Ti_3_AC_2_ (A = P, As, Cd) family. Since shorter bond lengths lead to strong bonding, the M–C slabs possess stronger bonds as compared to bonds in the M–A slabs. It should be noted that at 0 GPa, the C1–Ti4 bond (2.10523 Å) is 3.2% shorter than its counter bond C2–Ti1 (2.18472 Å) in Ti_3_PC_2_. This implies that the C1–Ti4 bond is stronger. Similar analyses on the A elements (A = P, As, Cd) reflect that the P1–Ti6 bond (2.48509 Å) is 3.0% and 19.9% shorter than the As1–Ti3 (2.56021 Å) and Cd1–Ti6 bonds (2.97993 Å), respectively, indicating that the P1–Ti6 bond is comparatively stronger than its counterparts, *i.e.*, As1–Ti3 and Cd1–Ti6. Table 6 discloses that the P2–Ti5 bond possesses a larger magnitude of bond stiffness, *i.e.*, 654 GPa, compared to that of the As2–Ti5 bond (552 GPa) but less than the bond stiffness calculated for Cd2–Ti5 (1193 GPa). Moreover, Table 6 lists the coefficients of the second order polynomial fit of relative bond lengths as a function of pressure for Ti_3_PC_2_, Ti_3_AsC_2_ and Ti_3_CdC_2_. Here it is noteworthy that the negative magnitudes of coefficient *C*_1_ and positive magnitudes for *C*_2_ reveal an increase in the deformation resistance to compression with increasing pressure. This result is obvious from Fig. 9, which illustrates the decreasing trend of bond length. However, some negative values of coefficient *C*_2_ demonstrate that an increase in the deformation resistance to compression with increasing pressure is relatively slow, as noticed particularly for the Ti2–Ti6 bond. In addition, it can be noticed that the relative stiffness (*i.e.*, the ratio of the bond stiffness of the weakest bond to that of the strongest bond) is greater than ½ for Ti_3_PC_2_ and Ti_3_AsC_2_ but lower than ½ for the Ti_3_CdC_2_ compound (Table 6). This means that Ti_3_PC_2_ and Ti_3_AsC_2_ are closer to typical ceramics, which possess low damage tolerance and fracture toughness.^72,75^ However, in the case of the Ti_3_CdC_2_ compound, a few unusual properties might be expected, such as its unusual stiffness of 4526 GPa for the Ti2–Ti6 bond.

**Table 5:** Bond lengths and bond population of Ti_3_AC_2_ (A = P, As, Cd)

Composite	Bonds				Bond length (Å)				
					0 GPa	5 GPa	10 GPa	15 GPa	20 GPa
Ti_3_PC_2_	C1–Ti4	C3–Ti6	C4–Ti5	C2–Ti3	2.10523	2.09254	2.07887	2.06590	2.05216
	C2–Ti1	C3–Ti2	C4–Ti1	C1–Ti2	2.18472	2.17020	2.15462	2.14117	2.12606
	P2–Ti5	P1–Ti6	P1–Ti3	P2–Ti4	2.48509	2.46707	2.45412	2.44168	2.43469
	Ti2–Ti6	Ti1–Ti5	Ti2–Ti4	Ti1–Ti3	2.95598	2.93971	2.92247	2.90698	2.88810
Ti_3_AsC_2_	C4–Ti5	C3–Ti6	C1–Ti4	C2–Ti3	2.08396	2.07373	2.06221	2.05094	2.04105
	C3–Ti2	C2–Ti1	C1–Ti2	C4–Ti1	2.17351	2.16077	2.14650	2.13346	2.12038
	Ti5–As2	Ti3–As1	Ti4–As2	Ti6–As1	2.56021	2.53600	2.52040	2.50360	2.49036
	Ti2–Ti6	Ti2–Ti4	Ti1–Ti5	Ti1–Ti3	2.92168	2.90907	2.89415	2.88085	2.86834
Ti_3_CdC_2_	C4–Ti5	C1–Ti4	C3–Ti6	C2–Ti3	2.05013	2.03233	2.02117	2.01164	2.00185
	C2–Ti1	C3–Ti2	C4–Ti1	C1–Ti2	2.17539	2.15182	2.13769	2.12577	2.11256
	Ti1–Ti5	Ti2–Ti6	Ti2–Ti4	Ti1–Ti3	2.89483	2.89334	2.88362	2.87191	2.85867
	Ti4–Cd2	Ti6–Cd1	Ti5–Cd2	Ti3–Cd1	2.97993	2.97423	2.93767	2.90332	2.87595

**Table 6:** Coefficients (*C*_1_, *C*_2_) of the second order polynomial fit of relative bond length as a function of pressure and bond stiffness (*k*) for Ti_3_AC_2_ (A = P, As, Cd)

Composite	Bonds	*C*_1_ × 10^−3^	*C*_2_ × 10^−5^	*k* (GPa)	Relative stiffness
Ti_3_PC_2_	C1–Ti4	−1.220	−0.190	820	0.88
	C2–Ti1	−1.360	0.124	735	0.79
	Ti2–Ti6	−1.070	−0.335	935	1.00
	P2–Ti5	−1.530	2.590	654	0.70
Ti_3_AsC_2_	C1–Ti4	−1.070	0.128	935	0.90
	C2–Ti1	−1.240	0.072	806	0.77
	Ti2–Ti6	−0.959	0.178	1043	1.00
	As2–Ti5	−1.810	2.310	552	0.53
Ti_3_CdC_2_	C1–Ti4	−1.180	0.774	847	0.19
	C2–Ti1	−2.090	3.350	478	0.11
	Ti2–Ti6	−0.221	−2.010	4525	1.00
	Cd2–Ti5	−0.838	−4.850	1193	0.26

### 3.4. Magnetic properties

**Fig. 10:**
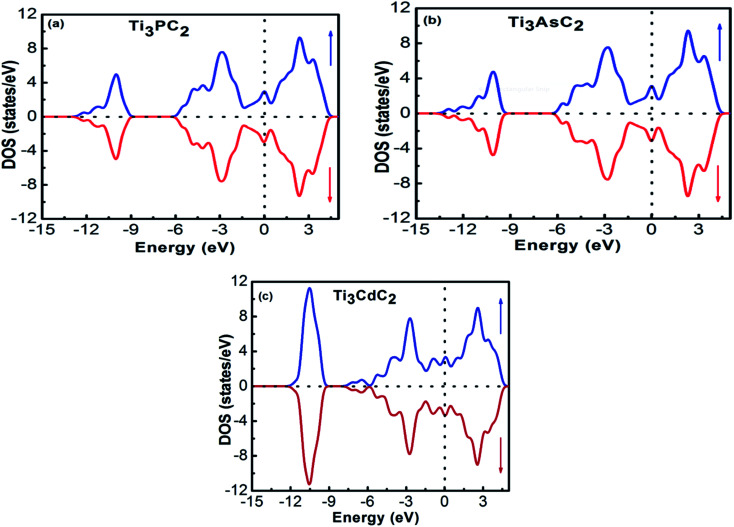
Spin-polarized DOS of (a) Ti_3_PC_2_, (b) Ti_3_AsC_2_ and (c) Ti_3_CdC_2_.

The interaction of the constituent elements of a solid and the crystal fields produces significant effects, which are duly recognized as magnetism.^76^ To account for these effects in the considered MAX compounds, the DFT + U functional was implemented. Regarding this, the spin-polarized DOS of Ti_3_PC_2_, Ti_3_AsC_2_ and Ti_3_CdC_2_ were estimated and are shown in Fig. 10(a)–(c). The Fermi level was adjusted at 0 eV. It is observed that the spin up ↑ states are exact replicas of the spin down ↓ states in each studied compound, assuring the antiferromagnetic behavior of these materials. Moreover, due to such a symmetric behavior of the spin up ↑ and spin down ↓ states, the net magnetic moment for all compounds is observed to be zero. The existence of few states at the *E*_F_ further endorses the metallic nature of the compounds. It is important to mention here that prior to this study, neither theoretical nor experimental observations regarding the magnetic properties of these materials have been reported.

**Fig. 11:**
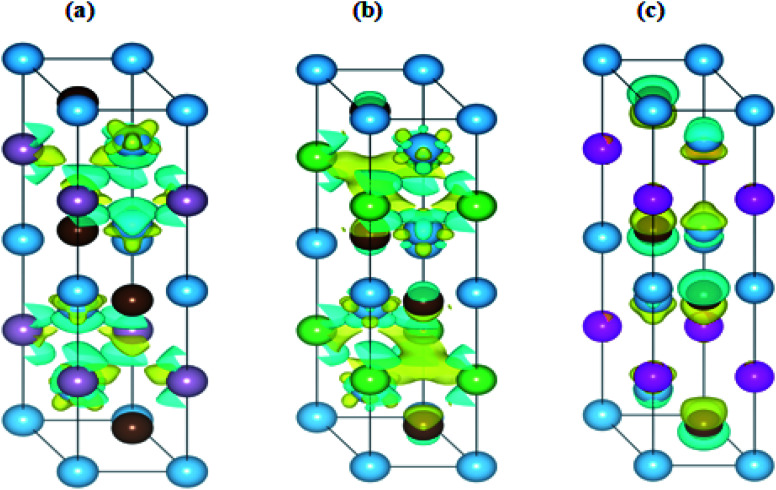
Iso-surface charge density calculations of (a) Ti_3_PC_2_, (b) Ti_3_AsC_2_ and (c) Ti_3_CdC_2_.

In Fig. 11(a)–(c), the charge difference calculation (isosurface charge density) plots clearly demonstrate the charge accumulation (yellow color) and charge depletion (cyan color). It has also been noticed from the charge density plots that most of the charge is either accumulated or depleted in between the interlayers and on the Ti atoms in the case of the Ti_3_PC_2_ and Ti_3_AsC_2_ composites, while in the case of Ti_3_CdC_2_, an inadequate amount of charge is accumulated and depleted on the C atoms. These results illustrate the antiferromagnetic behavior of the studied compounds.

## 4. Conclusions

In the present article, a DFT-based first-principles study has been carried out to investigate the structural, optoelectronic, mechanical and magnetic properties of Ti_3_AC_2_ (A = P, As, Cd). As no significant band gap exists between the valence and conduction bands, this fact depicts that the considered materials behave like conductors. The presence of DOS around the *E*_F_ describes the contribution of Ti 3d states to the conduction mechanism. The results pertaining to the elastic behavior indicated that these materials are not only thermally stable but mechanically stable as well. As per the data from Pugh's ratio, Ti_3_PC_2_ and Ti_3_AsC_2_ demonstrate a brittle nature, while Ti_3_CdC_2_ is ductile in nature. The values of relative stiffness greater than ½ reveal that Ti_3_PC_2_ and Ti_3_AsC_2_ are closer to typical ceramics, which possess low damage tolerance and fracture toughness. The magnitude of reflectivity reveals that these materials can be potential candidates for coating various devices to prevent solar heating. Moreover, the studied materials are dynamically stable and exhibit antiferromagnetic behavior. Henceforth, these MAX materials with hexagonal phases would be very appropriate potential candidates for coating applications.

## Conflicts of interest

There are no conflicts to declare.

## Supplementary Material
